# Evaluation of a peer intervention project in the hospital setting to improve the health-related quality of life of recently diagnosed people with HIV infection

**DOI:** 10.1186/s12955-023-02185-z

**Published:** 2023-09-13

**Authors:** M. J. Fuster-RuizdeApodaca, D. Pérez-Garín, V. Baceiredo, A. Laguía, J. García Carrillo, R. García, D. García

**Affiliations:** 1 Department of Social and Organizational Psychology. Psychology Faculty, Universidad Nacional de Educación a Distancia (UNED), Madrid, Spain; 2 Spanish Interdisciplinary AIDS Society, Madrid, Spain; 3 Infectious Diseases Unit, University Hospital V. Del Rocío, Seville, Spain; 4 Adhara Association, Seville, Spain; 5 Infectious Diseases Unit, University Hospital V. De Valme, Seville, Spain; 6 Infectious Diseases Unit, University Hospital V. Macarena, Seville, Spain

**Keywords:** HIV, Peer intervention, Antiretroviral therapy, Health-related quality of life, WHOQOL-HIV-BREF

## Abstract

**Purpose:**

This study aims to assess the impact of a peer intervention programme in the hospital setting to improve the health-related quality of life (HRQoL) of people recently diagnosed with HIV infection.

**Methods:**

A quasi-experimental single-group study with pre- and post-measurements was conducted. The peer intervention programme consisted of four sessions that took place at the following times: (1) the day of diagnosis, (2) the day when the results of the analyses were collected and ART (antiretroviral therapy) began, (3) one month after the start of ART, and (4) four months after the start of ART. The dependent variables were HRQoL and several of its psychological predictors. Change in the dependent variables was analysed through repeated measures, variance analysis and covariance analysis. Forty-three people with HIV participated in the intervention (40 men, mean age = 39.14).

**Results:**

A significant positive evolution was found in all the predictors of HRQoL, except avoidant coping (*p* < .05). A positive evolution was also found in all HRQoL dimensions (*p* < .05). There was a significant increase in CD4 cells/mm^3^ lymphocytes (*p* < .0001) and in the CD4/CD8 ratio (*p* < .001). The positive differential scores in the psychological health and social relationship dimensions influenced the increase in CD4 cells/mm^3^ lymphocytes (*p =* .012, *p =* .13). The increase in the social relations dimension score and overall health perception influenced the recovery of the CD4/CD8 ratio (*p =* .044; *p = .*068).

**Conclusions:**

Peer intervention improved the HRQoL of people recently diagnosed with HIV, and enhanced psychological health and social relationships covariate with their immunological recovery. This study represents an essential advance in evaluating peer intervention programmes for positive prevention.

Advances in antiretroviral treatment (ART) have increased life expectancy for people with HIV (PHIV). However, research shows that problems associated with HIV infection considerably impact these people’s health-related quality of life (HRQoL) [1, 2]. Adherence to ART and medical follow-up; health habits; or the management of psychological and social problems such as anxiety, depression, or HIV-associated stigma, among others, are essential factors that influence coping with the health process and HRQoL [3]. Supporting PHIV to deal with these situations is a critical element in infection management. Peer support is recognised as important for this purpose. The existing scientific data support the effectiveness of peer education in health programs, and national and international declarations recommend its use in different health fields. Peer intervention has essential advantages. Among them is that these people are a source of credible information for their peers. Also, the information they provide may be more influential than that of some professionals due to the identification process andtheir excellent knowledge of the characteristics and problems of their peers. In addition, peers function as positive role models and have physical and sociocultural access to their target population [4].

There are several areas where peer intervention is effective. A significant one is the provision of social support. Peers provide emotional support to address difficult situations, for example, by listening, sharing their stories, giving hope, and helping to increase self-esteem and the snormalisation of the process. They also provide instrumental support; that is, they assist with medical, bureaucratic, and care formalities or help to solve various needs. They also provide information and advice on topics such as adherence to treatment, healthy habits, health behaviours, etc. Finally, they are helpful in promoting affiliation through increasing contact with groups, other people, and social support networks [5].

Social support is a powerful tool to improve health as it enables social integration and has been shown to increase survival and longevity in various conditions, such as cancer. It decreases depression or susceptibility to infectious diseases [6]. Peer support also positively influences healthy habits and, therefore, primary, secondary, or tertiary prevention. In short, through social support, peer support can positively impact health by reducing isolation and feelings of loneliness, promoting healthy habits, and discouraging nonadaptive behaviours. It also encourages positive psychological states and individual motivation, providing information on behaviours and services that help well-being and prevent the risk of disease or its progression [7]. A systematic review published in 2011, which included 117 articles, confirmed the effectiveness of peer intervention in modifying risky sexual behaviours, attitudes, beliefs, and knowledge about HIV and reducing substance abuse [8]. Other systematic reviews, however, have yielded mixed results regarding the effectiveness of peer interventions for improving ART adherence, viral suppression, mortality, and several patient-reported outcomes [9, 10]. A more recent meta-analysis, including 20 randomised controlled trials, found that peer intervention significantly improved retention in care, adherence to antiretroviral therapy and viral suppression. However, evidence for other positive outcomes (antiretroviral therapy initiation, CD4 cell count, quality of life, and mental health) was promising but inconclusive [11]. A review published in 2022 shows that peer social support helps PHIV overcome the negative impact of both anticipated and internalised stigma, which is of utmost importance, as stigma is a known barrier to HIV treatment and care [12].

In Spain, few peer intervention programmes have been scientifically evaluated. To our knowledge, the only study published in Spain showed the effectiveness of peer intervention in promoting adherence to ART [13]. This study, involving 240 people with HIV, found that psychoeducational interventions carried out by peers obtained better results in adherence to ART and reduction of emotional distress than those conducted by healthcare professionals.

This study aims to assess the impact of a peer intervention program carried out in the hospital setting to improve the HRQoL of people with a new HIV diagnosis. In this way, the present study analyses the changes the intervention programme has brought about in the sample of participants over time. Likewise, the analysis of the effectiveness of intervention programmes is necessary in the field of prevention to promote evidence-based programmes.

## Methods

### Design and procedure

The intervention project was evaluated using a quasi-experimental single-group design and pre- and post-measurements. An intermediate evaluation measure was also administered.

The programme was conducted at the three university hospitals treating PHIV in Seville: Virgen del Rocío, Virgen Macarena, and Virgen de Valme. It was carried out between 2018 and 2020. Participants were offered peer interventions coinciding with the four scheduled visits usually attended by PHIV during the first year after diagnosis. Each session lasted an average of one hour.

Newly diagnosed PHIV were offered participation in the hospital’s peer programme by the Infectious Diseases Service healthcare professionals at each collaborating hospital. This participation consisted of attending the intervention sessions structured with the peer, coinciding with the four scheduled clinical visits. The first visit was held after the diagnosis to reduce its impact. The following held ten days after the treatment initiation and in the third and ninth months. The peer interventions were carried out with the users in a private consultation, integrated into the same Infectious Diseases Unit, along with the rest of the consultations of healthcare professionals who care for patients.

The programme sessions were conducted by three people with HIV (peer educators). These peers hold an 18-credit Official Professional Expert degree granted by UNED (Universidad Nacional de Educación a Distancia, Spain) for the education of peer educators to support PHIV. They also regularly attend training courses on key topics needed to provide qualified advice to PHIV regarding emotional, physical, and social well-being.

The questionnaire, including the study’s PROMs,was administered by the peers. The first questionnaire was administered after diagnosis, the second, fourth months after the start, and the third one a year after the intervention began. Healthcare professionals collected clinical data at each hospital from participants’ medical records.

The NGO Adhara develops the programme in close collaboration with the above-mentioned hospitals in which the intervention was carried out, the City Council, and the Sexually Transmitted Infections Center in Seville. Before the start of the intervention, we obtained consent from the heads of the Infectious Disease Units of each participating hospital. Before the beginningof the intervention, participants received sufficient information on the study’s objectives, and their written informed consent was collected. The Clinical Ethics Committees of the university hospitals Virgen Macarena and Virgen del Rocío approved the study protocol.

### Participants

Forty-three PHIV participated in the intervention (46 patients started the study, one of whom was a false positive for HIV and two changed their city of residence and hospital at the follow-up and were not able to continue the study). Most were men with a homosexual sexual orientation, with a mean age of slightly less than 40 years old, who had acquired HIV sexually. Over 37% had completed university studies, and nearly half worked regularly (Table 1). The sample size corresponded to almost 100% of new diagnoses in the study period. However, using the G-Power software, we tested the sample size required for an effect size *f* = 0.25, an α error = 0.05 and a power (1-β) = 0.95 to perform ANOVA with three intrasubject repeated measures. Results showed that the total sample required would be 44 people, yielding a critical value of *F* = 3.10.

**Table 1:** Sociodemographic and participation data

total N participants	43
N Questionnaires	
Initial measurement (baseline)	43
Intermediate measurement	30
Post measurement	34
Sociodemographic data	
Sex, n (%)	
Man	40 (93)
Woman	3 (7)
Age (M ± SD)	39.14 ± 10.18
Educational level	
No studies	3 (7)
Primary	10 (23.3)
Secondary	14 (32.6)
University	16 (37.2)
Work situation	
Working with contract	21 (48.8)
Working without contract	4 (9.3)
Doesn’t work	14 (32.6)
Occupational disability	1 (2.3)
No reply	3 (7)
Sexual orientation	
Heterosexual	8 (18.6)
Homosexual	25 (58.1)
Bisexual	7 (16.3)
Prefers not to respond	3 (7)
Transmission pathway	
Sexual relation	41 (95.3)
Doesn’t know	2 (4.7)
ART Start Time (days, M ± SD)	20.47 ± 18.04

### Variables and measurements

#### Independent variable: intervention program

The study’s independent variable was the peer intervention in the hospital setting. This intervention consisted of four sessions that took place at the following times. The first session was on the day of diagnosis, the second was on the day when the results of the analyses were collected and ART began, the third was one month after the start of ART, and the fourth was four months after the start of ART. The sessions had semi-structured content (Table 2), although they were adapted to fit the needs expressed by the recipients and concerning the moment they were undergoing.

**Table 2:** Structure and contents of the intervention programme

Session	Temporal moment	Objective	Components	Assessment
First	Diagnosis day	Collect baseline data for intervention impact assessment		First administration of questionnaires on the quality of life and its predictors Collect clinical baseline data
		Promote acceptance of the diagnosis	Anxiety reduction Promote calmness and reassure Reduce guilt	
		Provide information on HIV	Basic information on HIV and AIDS HIV transmission pathways	
		Promote sexual prevention	Information about sexual practices Information about sexual partners Information about sexually transmitted infections (STI) and their prevention	
		Reduce the social impact of HIV and boost social support	Family, social networks, and partner Sharing the serological status Affective-sexual intercourse	
		Provide instrumental social support.	Managing visits without appointments Reporting diagnostic start-up testing protocols Presentation of the clinical staff	
Second	Collect analytical results Start of ART	Boost self-esteem and empowerment	Sexual acceptance Self-discrimination Self-stigma Mourning	
		Promote prevention and healthy habits	Role of viral load in HIV transmission Valuation of drug use Damage reduction Substance and consumption ART-drug interactions	
		Boost retention in care and adherence to ART	Address the healthcare professionals-patient relationship Interpretation of analytics Information about ART Information about undetectability Provide tools to improve adherence Basic information on side effects Healthy habits	
		Provide instrumental social support	Accompaniment in the collection of ART Management of the next appointment	
Third	One month after the start of ART	Boosting retention in care and adherence to ART	Address the personal health-patient relationship Viral load information Analysis of perceived side effects Analyse perceived barriers to adherence and providing tools Alternative medicine Healthy habits	
		Reduce the social impact of HIV and boost social support	Analysis of difficulties at work, family, social networks, and partner Affective-sexual intercourse Tools to cope with stigma and discrimination	
		Provide instrumental social support.	Accompaniment to the pharmacy	
Fourth	Four months after the start of ART	Provide information on gender and transsexuality aspects	Pregnancy Menopause Reproduction Hormones Vaccination	
		Provide information on the rights of people with HIV (PHIV)	Legal aspects Work aspects	
		Improve psychological health	Assess the acceptance of sexuality, self-discrimination, and self-stigma Valuation of mourning elaboration Obstacles	
		Provide information about the health process.	Comorbidities	
		Provide instrumental social support	ART pickup and/or shipping Manage visits without appointments Follow-up of patients missing ART collection	
		Perform the appropriate referrals.	Hospital-social worker Hospital-pharmacy Hospital-psychology Hospital-others Specialised associations Services of the NGO Adhar: psychological care, job orientation, support groups, workshops	
		Assessment of the impact of the intervention programme		Second administration of questionnaires of quality of life and its predictors
Fifth	12 months from diagnostic day and baseline data collection	Assessment of the impact of the intervention programme		Last administration of questionnaires of quality of life and its predictors Data collection

#### Dependent variables: repeated measures

##### HRQoL

The validated Spanish version of the WHOQOL-HIV-BREF (the HIV version of the World Health Organisation Quality of Life Assessment-Bref) was used [14]. This patient-reported outcome measures (PROM) measures six dimensions of quality of life: physical health, psychological health, level of independence, social relations, environment, and spirituality. In addition, it measures the perception of overall health and quality of life through two items.

##### Psychological predictors of HRQoL

The ScreenPLHIV Questionnaire was used [15]. This PROM comprises 63 items covering 23 protective or risk facets of quality of life. We used 21 facets in the present study (*protective facets*: social support, self-esteem, problem-focused coping, positive re-evaluation, optimism, personal meaning, change in personal values, personal autonomy, activism, healthy habits, and disease information; *risk facets*: emotional loneliness, sexual dissatisfaction, negative disease representation, avoidant coping, economic problems, the experience of rejection, perception of rejection, internalised stigma, stress due to HIV, and depressive mood) as two of them were linked to ART, and the participants entered the project when diagnosed and had not yet started ART.

##### Clinical markers

We collected the following immunological and virological markers of HIV infection: CD4 cells/mm^3^, CD4/CD8 ratio, and copies of basal viral load one year from the start of the intervention.

Sociodemographic data and other clinical variables (diagnostic date, ART start date) were also collected.

### Data analysis

First, we performed an exploratory analysis to detect missing, atypical, or extreme data, and to ensure that the statistical assumptions of multivariate analysis techniques were met. The following analyses were performed using the thirty-three paired questionnaires obtained. First, we evaluated if there were any differences in the sociodemographic variables. We did not find anyone. Next, to assess the change in psychosocial dependent variables (HRQoL and its predictors), repeated measures analysis of variance (ANOVA; mixed model or split-plot) was performed. Next, to study the association that the change in HRQoL predictors had on the evolution of each of its dimensions in the post-intervention measures, repeated measures analysis of covariance (ANCOVA) was carried out. Differential scores (baseline measurement minus end-of-intervention measurement) were included as covariates in the protective and risk facets of HRQoL. Also, to control the effect of health improvement on changes in the dimensions of HRQoL, ANCOVA was performed, including in the models the differential scores on the immune markers and viral load obtained across the period.

The evolution of the immune markers and viral load was analysed through Student’s *t*-test for related samples and confirmed with the Wilcoxon non-parametric test. We also performed an ANCOVA, including the CD4 cells/mm^3^ lymphocyte count and the CD4/CD8 ratio as dependent variables and the differential scores in HRQoL dimensions and its psychological predictors as covariates.

The data were analysed using the SPSS-PC Social Sciences statistical program for Windows (v.22.0).

## Results

### Evolution of HRQoL and its predictors

First, we analysed the change between the three repeated measures of the battery of HRQoL predictors and the dimensions of HRQoL collected from the participants (*n* = 30). Concerning the predictors of HRQoL, a significant positive evolution was found in almost all facets except for avoidant coping. The largest effect sizes were observed in decreased dissatisfaction with sexuality, internalised stigma, HIV-related stress, depressive mood, and negative HIV representation. Also, increased social support, optimism, and problem-focused coping showed remarkable effect sizes. Decreases in emotional loneliness and the experience of rejection, increased information about HIV, and positive re-evaluation of HIV were observed with a moderate effect size. Lastly, although the effect size was lower, there was a positive change in personal values and personal autonomy, and a decrease in the perception of rejection and economic problems (Table 3).

**Table 3:** Results of the repeated measures ANOVA of health-related quality of life predictors (ScreenPLHIV)

	Measurement 1		Measurement 2		Measurement 3		F(2, 28) (p)	ɳ^2^
	Mean	SD	Mean	SD	Mean	SD		
**Protective facets**								
Social support^b^	61.65	24.37	67.89	26.38	80.39	17.92	15.666 (p < .0001)	0.528
Self-esteem^a^	63.70	24.49	74.58	21.77	84.18	15.24	12.943 (p < .0001)	0.480
Problem-focused coping ^a^	73.03	19.92	83.76	16.22	88.63	11.94	13.569 (p < .0001)	0.492
Positive re-evaluation^c^	47.54	26.72	70.79	26.42	77.71	25.59	8.33 (0.001)	0.373
Optimism^a^	67.25	21.95	81.81	15.75	87.78	9.86	18.335 (p < .0001)	0.567
‘Personal Meaning’^a^	55.10	20.45	71.61	20.10	78.00	18.12	9.627 (0.001)	0.407
Change in personal values^b^	54.61	27.70	56.95	31.83	70.63	26.61	6.594 (0.005)	0.320
Personal autonomy^b^	68.35	21.59	76.46	22.38	83.23	19.09	6.673 (0.004)	0.323
Activism^e^	27.99	26.61	33.03	26.11	37.43	26.31	2.705 (0.084)	0.162
Healthy Habits^d^	69.30	22.91	81.47	16.77	86.20	15.92	12.790 (p < .0001)	0.477
Disease Information^b^	57.23	23.95	65.75	25.08	74.41	24.22	10.062 (0.001)	0.418
**Risk facets**								
Emotional loneliness^a^	41.78	28.18	27.49	25.51	17.33	21.35	11.024 (p < .0001)	0.450
Sexual dissatisfaction^a^	65.57	31.04	35.59	27.54	15.80	19.38	39.605 (p < .0001)	0.739
Negative disease representation^a^	67.05	23.46	49.61	16.64	41.84	14.94	15.522 (p < .0001)	0.526
Avoidant coping	57.98	27.66	51.45	29.86	49.88	31.17	1.010 (0.378)	0.070
Economic problems^a^	36.01	29.02	16.73	22.03	11.83	19.86	8.417 (0.001)	0.384
Experience of rejection^d^	27.58	24.93	13.03	17.77	9.55	15.62	9.150 (0.001)	0.413
Perception of rejection^b^	74.22	26.03	70.34	55.29	52.77	23.57	8.328 (0.002)	0.382
Internalised stigma^a^	55.95	31.71	33.65	28.16	18.47	18.59	25.692 (p < .0001)	0.656
Stress due to HIV^a^	49.15	32.79	22.03	21.32	11.35	18.06	22.448 (p < .0001)	0.616
Depressive mood^a^	42.42	26.19	21.13	23.09	9.74	16.84	20.928 (p < .0001)	0.599

^a^Significant differences between all pairs of measurements (p < .05). ^b^Significant differences between all pairs of measurements except between 1 and 2 (p < .05). ^c^Significant differences between all pairs of measures except between 1 and 3 (p < .05). ^d^Significant differences between all pairs of measurements except between 2 and 3 (p < .05). ^e^Marginally significant difference between measurements 1 and 3 (< 0.10)

**Fig. 1:**
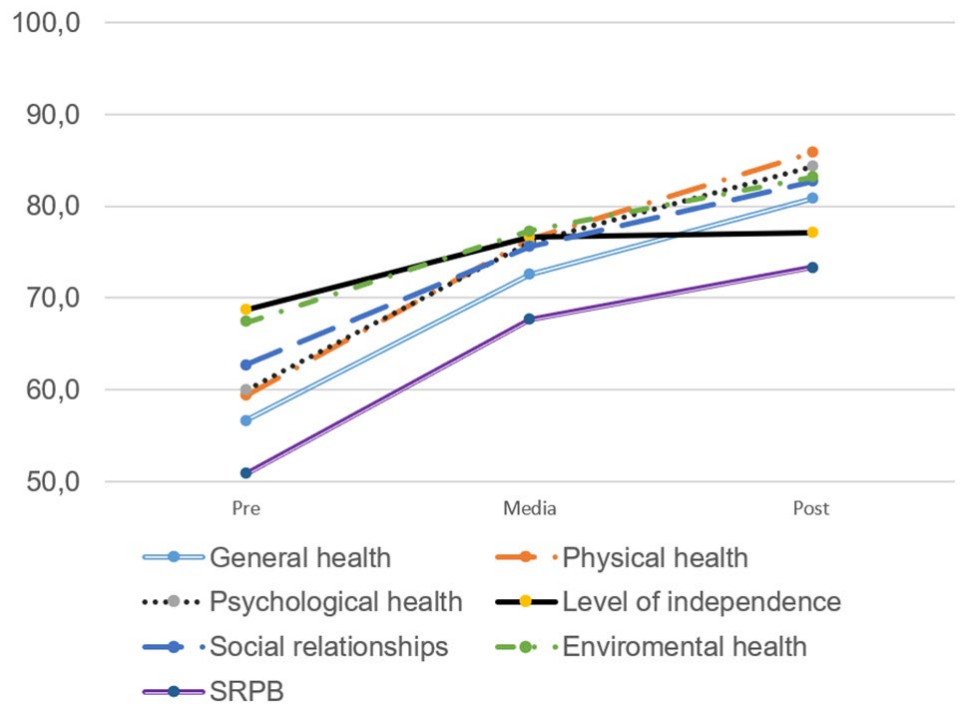
Evolution of the dimensions of HRQoL during the peer intervention. SRPB: spirituality, religion, and personal beliefs

Regarding HRQoL, the results showed a significant positive evolution in all its dimensions; participants had higher scores in overall health, physical health, psychological health, level of independence, social relations, environmental health, and spirituality (Table 4; Fig. 1).

**Table 4:** Results of the repeated-measures ANOVA of health-related quality of life (WHOQOL-HIV-Bref)

	Measurement 1		Measurement 2		Measurement 3		F(2, 28) (p)	ɳ^2^
	Mean	SD	Mean	SD	Mean	SD		
General health^a^	56.66	20.95	72.50	19.25	80.83	16.65	26.976 (p < .0001)	0.658
Physical health^a^	59.37	17.73	76.45	14.08	85.83	14.39	42.756 (p < .0001)	0.753
Psychological health^a^	59.90	15.89	76.00	12.62	84.33	12.71	46.543 (p < .0001)	0.769
Level of independence^b^	68.75	15.04	76.66	15.65	77.08	13.76	5.343 (p = .011)	0.276
Social relations^a^	62.70	21.92	75.60	14.62	82.70	12.99	16.692 (p < .0001)	0.544
Environmental health^a^	67.31	11.15	77.29	11.01	83.23	9.83	31.477 (p < .0001)	0.692
Spiritual, religion and personal beliefs dimension (SRPB)^a^	50.90	21.13	67.71	19.97	73.33	18.63	16.394 (p < .0001)	0.539

^a^Significant differences between all pairs of measurements. ^b^Significant differences between all pairs of measurements except between 2 and 3

### Covariates of the positive evolution in the HRQoL dimensions

We analysed the association of the differential scores in the quality of life predictor facets with the change in each of the dimensions of HRQoL after the intervention. Concerning the protective facets of quality of life, differential scores in disease information, personal autonomy, and positive re-evaluation were significantly associated with positive developments in overall health perception, *F(2,17)* = 3.587, *p =* .050, ɳ^2^ = 0.297; *F(2,17) =* 11.720, *p(2,17) =* 0.001, ɳ^2^ = 0.580; *F(2,17) =* 6.435, *p(2,17) =* 0.008, ɳ^2^ = 0.431, respectively. Also, the differential problem-focused coping score was associated with the positive evolution of psychological health, *F(2,17) =* 3.402, *p =* .057, ɳ^2^ = 0.286. The differential score in social support was associated with a positive change in the spirituality dimension, *F(2, 17) =* 3.863, *p =* .041, ɳ^2^ = 0.312.

Regarding the risk facets of quality of life, it was observed that the decrease in depressive mood was associated with a positive change in overall health perception, *F(2,17) =* 3.879, *p =* .041, ɳ^2^ = 0.313. The decrease in the perception of rejection was associated with the change in physical health, *F(2,17) =* 3.686, *p =* .047, ɳ^2^ = 0.303. Decreased dissatisfaction with sexuality was associated with the improved social relations dimension, *F(2,17) =* 3.201, *p =* .066, ɳ^2^ = 0.274. The decrease in emotional loneliness was associated with a positive change in the environmental health dimension, *F(2,17) =* 7.183, *p =* .005, ɳ^2^ = 0.458. Finally, the decrease in the negative representation of the disease and the perception of rejection was associated with a positive change in the spiritual dimension of quality of life, *F(2,17) =* 6.022, *p =* .011, ɳ^2^ = 0.415; *F(2,17) =* 4.853, *p =* .022, ɳ^2^ = 0.363, respectively.

The differential scores on the immune markers and viral load obtained over time were included as covariates in the model. It was observed that the increase in CD4/CD8 ratio interacted significantly with the increase in the score of the social relations dimension, *F(2,21) =* 4.846, *p =* .019, ɳ^2^ = 0.316.

### Evolution in the Immunological and virological status: The association of improvement in HRQoL and its predictors

The participants’ immunological status improved during the intervention and assessment period. A significant increase was observed in CD4 cells/mm^3^ lymphocytes (Mbaseline = 377.91 ± 226.19 vs. Mpost = 642.45 ± 311.01; t = − 6.863, p < .0001; Cohen’s *d* = − 0.923) and in the CD4/CD8 ratio (Mbaseline = 0.51 ± 0.31 vs. Mpost = 0.88 ± 0.47, *t* = − 4.713, Cohen’s *d* = − 0.887).

#### HRQoL

The ANCOVA results showed that positive differential scores in the psychological health and social relationship HRQoL dimensions influenced the increase in CD4 cells/mm^3^ lymphocytes, *F(1,21) =* 7.554, *p =* .012, ɳ^2^ = 0.265 and *F(1,21) =* 7.350, *p = .*013, ɳ^2^ = 0.259, respectively. Besides, it was found that the increase in the score of the social relations dimension and overall health perception influenced the recovery of the CD4/CD8 ratio, *F(1,21) =* 4.586, *p =* .044; ɳ^2^ = 0.179 and *F(1,21) =* 3.712, *p = .*068, ɳ^2^ = 0.150, respectively. Subsequent ANCOVAs were then performed with quality of life predictors as covariates. About the protective facets, we observed that the improvement in self-esteem and optimism was positively and significantly associated with the increase in the CD4/CD8 ratio, *F(1,23) =* 4.819, *p =* .039, ɳ^2^ = 0.224; *F(1,24) =* 3.298, *p =* .082, ɳ^2^ = 0.121, respectively. Regarding the risk facets of quality of life, it was observed that the decrease in score of internalised stigma was significantly associated with the increase of CD4 cells/mm^3^, *F(1,20) =* 8,610, *p =* .008, ɳ^2^ = 0.301, and also that the decrease in the experience of rejection was marginally significantly associated with the increase of the CD4/CD8 ratio, *F(1,24) =* 3.598, *p =* .070, ɳ^2^ = 0.130.

As for the viral load, we confirmed that the reduction in the median (Mbaseline = 1,122.240, range 2,380 to 11,000.000; Mpost = 20, range 20 to 6,250) of the viral load was significant (*p* < .0001). Thus, at baseline, 100% of patients had a detectable viral load, whereas, at the last measurement taken at the end of the programme, 68.3% had a viral load < 20 copies mm^3^, 22% < 50 copies mm^3^, and only 9.8% still had a detectable viral load.

It was observed that none of the quality of life dimensions was associated with a decreased viral load. The facets of the different protective or risk facets of quality of life were not associated with a reduced viral load, except for a marginally significant influence of the differential score of the economic problems facet, *F(1,24) =* 4.157, *p =* .053, ɳ^2^ = 0.148.

## Discussion

This study aimed to evaluate the effectiveness of a peer intervention programme in a hospital setting to improve the HRQoL of patients with a new HIV diagnosis. According to evidence of the determinants and predictors of the HRQoL of PHIV [2, 3], the contents of the intervention covered critical aspects for the adequate self-management of the process of living with HIV [16]. Thus, the intervention programme included medical, behavioural, and emotional management content. In addition, key self-management health skills such as relationships with healthcare professionals or resource utilisation were also addressed [16].

The results showed a positive evolution in all the dimensions of participants’ HRQoL. There was a large change in health perception, physical, psychological, and environmental health. The difference was moderate to high in the social relationships and spiritual dimensions, the latter of which measures existential issues relevant to the PHIV process, such as stigma, concern about the future, and death. In line with other studies, this was the dimension where participants scored the lowest [14].

A positive change in most measured quality of life predictors was also found after the intervention. These changes stand out for their size: increased perceived social support, self-esteem, problem-focused coping strategies, optimism, healthy habits, or disease information. We also highlight the reduction in risk facets of quality of life, such as dissatisfaction with sexuality, negative disease representation, perceived and internalised stigma, depressive mood, and emotional loneliness. Improvement in all these aspects may be related to being included directly among the components of the intervention, or they may be effects of providing various sources of social support by peers [5]. This intervention offered informational, emotional, instrumental, social, and affiliative support. There is abundant evidence to show that social support improves health and HRQoL through increased social integration; healthy habits; primary, secondary, and tertiary prevention; and the reduction of barriers to care, among other direct mechanisms [6, 17]. The results showed that the positive change in many of these quality of life predictors was associated with improved scores on HRQoL dimensions.

Participants in the programme significantly improved their immune status after one year of diagnosis, and most of them achieved virological suppression. This result was not directly related to the peer intervention programme, but it is a direct consequence of the medical intervention, primarily, ART initiation. However, the results showed that improved psychological health and social relationships resulting from peer intervention was associated with immune recovery. This result is consistent with evidence documenting the effects of psychological well-being and social support on health [7, 17, 18].

This peer intervention assessment study in the hospital setting has some relevant strengths. The evidence fills research gaps in the area. Thus, it increases the evidence of a peer intervention’s effectiveness in positive prevention, as few studies have been published in this area [8–12]. It also incorporates objective health measures into the evaluation, as peer intervention efficacy studies, including biological markers, are scarce [8, 19]. The intervention wa structured and designed according to the existing evidence on the protective and risk facets of quality of life [2, 20]. Moreover, the peers involved have homogeneously regulated training. Homogeneous training makes their role more uniform, increases their effectiveness, and raises its value for stakeholders [7].

The present study results pose a signific contribution to establishing the effectiveness of peer intervention for PHIV, especially since this intervention shows substantial improvements in variables in which a recent meta-analysis [11] found that the existing evidence still showed a low quality, such as mental and physical quality of life (our data show a significant improvement in those scores and every other score measured by WHOQOL-HIV-BREF), depressive mood, and CD4 cell count.

The study also presents some limitations. First, the evaluation design is not experimental, as it has no control or non-equivalent control group. The literature recognises the difficulty of using experimental designs in programme evaluation [20]. In the case of this programme, it should be noted that virtually all the newly diagnosed people are referred to the educator and benefit from the programme, so it is not possible to have control cases. Having a quasi-control group in the study city was also impossible because all of the hospitals treating people with HIV in Seville participated in the intervention. Adding pre-intervention measures to design a model to allow for regression discontinuity analysis was also impossible, although it would have reduced threats to validity. This is because newly diagnosed people are automatically referred to peer educators. Future studies should explore overcoming these limitations, for example, by pairing a quasi-control group in another city with a similar health and cultural context or by establishing a longer longitudinal follow-up. However, we tried to reduce the threat to the validity of such a design by measuring the PROMs in three intervention moments, pre and post-defined periods, and an intermediate -one-fourth months after the start of ART. The results show changes between the three evaluation measures in most dependent variables. However, not all people included in the study completed all three measures, reducing the sample size even more. This occurred because of the fatigue of answering questionnaires but mainly because of circumstances linked to Covid-19, which prevented the face-to-face collection of the post-intervention questionnaires. Nevertheless, post-hoc analyses showed that with a sample of 34 participants (those who completed the third measure), the critical value of *F* = 3.13 and power (1- β) = 0.88. Most *F* values we found were higher than the critical *F*. In addition, the scores obtained in the HRQoL dimensions are higher than the averages existing in PHIV in Spain [14]. Thus, we consider these results acceptable, given the impact of the pandemic on the study methodology and the relevance of the intervention.

In summary, this study represents an important advance in evaluating peer intervention programmes for positive prevention in the hospital setting. Due to resource and knowledge constraints, few NGOs rigorously evaluate these programmes. Despite the design limitations, the results have shown the usefulness of the intervention due its potential improvement in the HRQoL of recently diagnosed PHIV. The increase of positive protective factors and the reduction of risk facets, direct or indirect, product of the peer intervention was associated with this improvement.

## Data Availability

This study’s dataset has been made publically available in figshare.com, and is accessible through the following link: https://figshare.com/articles/dataset/Data_Set_Adhara_Peer_Intervention/21163825.

## References

[1] Lazarus JV, Safreed-Harmon K, Barton SE, Costagliola D, Dedes N, del Valero A (2016). Beyond viral suppression of HIV – the new quality of life frontier. BMC Med.

[2] Ballester-Arnal R, Gómez-Martínez S, Fumaz CR, González-García M, Remor E, Fuster MJ (2016). A spanish study on psychological predictors of quality of life in people with HIV. AIDS Behav.

[3] Degroote S, Vogelaers D, Vandijck DM (2014). What determines health-related quality of life among people living with HIV: an updated review of the literature. Archives of Public Health.

[4] Simoni JM, Franks JC, Lehavot K, Yard SS (2011). Peer interventions to promote health: conceptual considerations. Am J Orthopsychiatry.

[5] Dutcher MV, Phicil SN, Goldenkranz SB, Rajabiun S, Franks J, Loscher BS (2011). Positive examples: a bottom-up approach to identifying best practices in HIV care and treatment based on the experiences of peer educators. AIDS Patient Care STDs.

[6] Seeman TE (1996). Social ties and health: the benefits of social integration. Ann Epidemiol.

[7] Dennis CL (2003). Peer support within a health care context: a concept analysis. Int J Nurs Stud.

[8] Simoni JM, Nelson KM, Franks JC, Yard SS, Lehavot K (2011). Are peer interventions for HIV efficacious? A systematic review. AIDS Behav.

[9] Boucher LM, Liddy C, Mihan A, Kendall C (2020). Peer-led self-management interventions and adherence to antiretroviral therapy among people living with HIV: a systematic review. AIDS Behav.

[10] Genberg BL, Shangani S, Sabatino K, Rachlis B, Wachira J, Braitstein P (2016). Improving engagement in the HIV care cascade: a systematic review of interventions involving people living with HIV/AIDS as peers. AIDS Behav.

[11] Berg RC, Page S, Øgård-Repål A (2021). The effectiveness of peer-support for people living with HIV: a systematic review and meta-analysis. PLoS ONE.

[12] Øgård-Repål A, Berg RC, Fossum M (2021). Peer support for people living with HIV: a scoping review. Health Promot Pract.

[13] Ruiz I, Olry A, López M, Prada JL, Causse M (2010). Prospective, randomised, two-arm controlled study to evaluate two interventions to improve adherence to antiretroviral therapy in Spain. Enfermedades Infecciosas y Microbiología Clínica.

[14] Fuster-RuizdeApodaca MJ, Laguía A, Safreed-Harmon K, Lazarus JV, Cenoz S, Amo D (2019). Assessing quality of life in people with HIV in Spain: psychometric testing of the spanish version of WHOQOL-HIV-BREF. Health Qual Life Outcomes.

[15] Remor E, Fuster MJ, Ballester-Arnal R, Gómez-Martínez S, Fumaz CR, González-Garcia M (2012). Development of a new instrument for the assessment of psychological predictors of well-being and quality of life in people with HIV or AIDS. AIDS Behav.

[16] Lorig KR, Holman H (2003). Self-management education: history, definition, outcomes, and mechanisms. Ann Behav Med.

[17] Cobb S (1976). Social support as a moderator of life stress. Psychosom Med.

[18] Logie C, Gadalla TM (2009). Meta-analysis of health and demographic correlates of stigma towards people living with HIV. AIDS Care.

[19] Enriquez M, Cheng AL, McKinsey D, Farnan R, Ortego G, Hayes D (2019). Peers keep it real: re-engaging adults in HIV care. J Int Association Providers AIDS Care.

[20] Matt GE, Cook TD. Threats to the validity of generalized inferences. In: Cooper H, Hedges LV, Valentine JC, editors. The handbook of research synthesis and meta-analysis. 2nd ed. Russel Sage Foundation; 2009. pp. 537–60.

